# Disappearance of the Defining Organized Ultrastructural Lesion in Chronic Lymphocytic Leukemia-Associated Monoclonal Immunotactoid Glomerulopathy After Ibrutinib Therapy

**DOI:** 10.1016/j.ekir.2026.106643

**Published:** 2026-06-03

**Authors:** Shota Shibuki, Maki Yoshihara, Makoto Fukuda, Aya Nawata, Hiroo Katsuya, Dedong Kang, Kunio Kawanishi, Yayoi Ogawa, Shigehisa Aoki, Shinya Kimura, Motoaki Miyazono

**Affiliations:** 1 Division of Nephrology, Department of Internal Medicine, Faculty of Medicine, Saga University, Japan; 2 Department of Pathology and Oncology, University of Occupational and Environmental Health, Kitakyushu, Japan; 3 Division of Pathology, Department of Pathology and Microbiology, Faculty of Medicine, Saga University, Saga, Japan; 4 Department of Anatomy, Showa Medical University School of Medicine, Japan; 5 Department of Pathology, Sapporo Tokushukai Hospital, Sapporo, Hokkaido, Japan; 6 Division of Hematology, Respiratory Medicine and Oncology, Department of Internal Medicine, Faculty of Medicine, Saga University, Japan

**To the Editor:**

A 61-year-old man was referred for nephrotic syndrome with a urine protein-to-creatinine ratio of 5.97 g/gCr, serum albumin of 2.6 g/dl, and serum creatinine of 0.71 mg/dl (Figure 1a, Supplementary Table S1). Initial monoclonal studies were nondiagnostic. Kidney biopsy showed mild mesangial matrix expansion (Figure 1c) and glomerular basement membrane thickening with focal spikes and bubbling (Figure 1d). Immunofluorescence demonstrated segmental IgG1-κ restriction (Figure 1g, i, Supplementary Table S2), and electron microscopy revealed organized fibrillary-to-microtubular deposits measuring approximately 22 nm, with focal parallel alignment (Figure 1e, f). Laser microdissection–liquid chromatography–mass spectrometry of a limited glomerular sample detected more κ light chain variable region–derived peptides than λ-derived peptides, consistent with the κ-dominant immunofluorescence pattern (Supplementary Table S3). Although the microtubular substructures were narrower than those typically described in immunotactoid glomerulopathy, negative DNAJB9 staining argued against fibrillary glomerulonephritis and, together with the monoclonal staining pattern and ultrastructural features, supported a diagnosis of monoclonal immunotactoid glomerulopathy or glomerulonephritis with organized microtubular monoclonal Ig deposits.^1^, ^2^, ^3^

**Figure 1:**
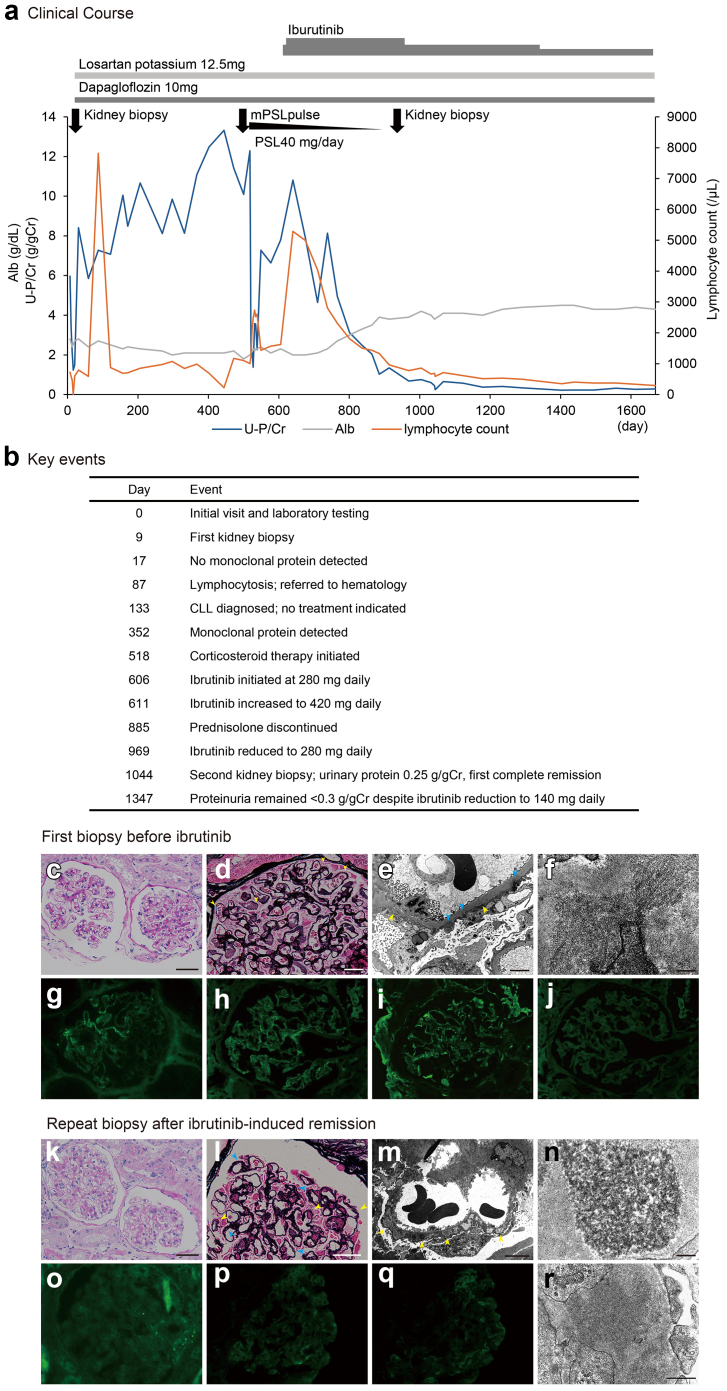
Clinical course and kidney biopsy findings before and after ibrutinib therapy. (a) Serial changes in urinary protein excretion, expressed as the urine protein-to-creatinine ratio (U-P/Cr), serum albumin, and lymphocyte count are shown from the initial visit (day 0). The timing of kidney biopsies, corticosteroid therapy, and ibrutinib therapy is indicated. Conservative treatment with losartan potassium and dapagliflozin was initiated after the initial evaluation. Methylprednisolone pulse therapy followed by oral prednisolone was started after worsening proteinuria. Ibrutinib was initiated on day 606 and subsequently adjusted according to the clinical course. Complete remission was achieved by the time of the second kidney biopsy on day 1044, and proteinuria remained at or below 0.3 g/gCr thereafter. (b) Key clinical events are summarized chronologically. No bleeding events or other clinically significant adverse events attributable to ibrutinib were observed during treatment. (c–j) Kidney biopsy findings before ibrutinib. (k–r) Repeat kidney biopsy after ibrutinib-induced remission. Findings from the first biopsy (c–j) and repeat kidney biopsy after ibrutinib-induced remission (k–r) are shown. (c) Periodic acid–Schiff (PAS) stain from the first biopsy shows mild mesangial matrix expansion and slight thickening of the glomerular capillary walls without significant hypercellularity. (d) Periodic acid–methenamine silver (PAM) stain from the first biopsy demonstrates focal spike formation and bubbling of the glomerular basement membrane (yellow arrowheads). (e) Electron microscopy from the first biopsy shows organized electron-dense deposits (cyan arrowheads), with focal podocyte foot process effacement (yellow arrowheads). (f At higher magnification, the deposits show fibrillary-to-microtubular substructure, measuring approximately 22 nm in diameter. (g) IgG1 staining in the first biopsy shows segmental granular deposits along the glomerular basement membrane. IgG2, IgG3, and IgG4 were negative (data not shown). (h) IgM staining in the first biopsy shows global capillary wall deposits. (i) κ light chain staining in the first biopsy shows segmental glomerular basement membrane deposits. (j) λ light chain staining in the first biopsy is negative. (k) PAS stain from the repeat kidney biopsy shows mild mesangial matrix expansion and slight thickening of the glomerular capillary walls. (l) PAM stain from the repeat kidney biopsy demonstrates delicate spike and bubbling formation (yellow arrowheads), with focal and segmental double contours in some capillary loops (cyan arrowheads). (m, n, r) Electron microscopy from the repeat kidney biopsy shows numerous intramembranous electron-lucent deposits within the glomerular basement membrane, consistent with Ehrenreich–Churg stage IV change. At higher magnification, the intramembranous deposits in the repeat biopsy show loss of the fibrillary-to-microtubular substructure observed in the first biopsy. (o) IgG1 staining in the repeat kidney biopsy shows only trace, segmental granular staining along the glomerular capillary walls. (p) κ light chain staining in the repeat kidney biopsy shows only trace, segmental staining. (q) λ light chain staining in the repeat kidney biopsy shows similar trace, segmental staining. In contrast to the first biopsy, repeat immunofluorescence showed no IgG subclass predominance or light chain restriction. Scale bars: c and k, 50 μm; d and l, 25 μm; e, 2 μm; f, 200 nm; m, 5 μm; n, 200 nm; r, 500 nm. Original magnification: g–j and o–q, ×400.

Because proteinuria transiently improved, conservative therapy was initially chosen.

During follow-up, chronic lymphocytic leukemia was diagnosed on day 133 based on sustained B-cell lymphocytosis and a CD5+/CD19+/CD20+/CD23+ immunophenotype. Although the patient had Rai stage 0 or Binet stage A disease without conventional hematologic indications for treatment, proteinuria worsened and serum immunofixation subsequently detected IgMκ, indicating ongoing kidney injury attributable to the clone. After steroid therapy failed to induce sustained remission, ibrutinib was initiated, leading to complete remission with reduction of urinary protein excretion to < 0.3 g/gCr.

Repeat kidney biopsy after remission showed only trace residual glomerular staining without the previous IgG subclass or light-chain restriction (Figure 1o–q, Supplementary Table S2). Electron microscopy demonstrated disappearance of the defining organized microtubular substructure, with residual electron-lucent intramembranous deposits (Figure 1m, n, and r), indicating morphologic regression after suppression of pathogenic immunoglobulin production. However, the increase in globally sclerotic glomeruli suggested an irreversible component of chronic injury, likely reflecting delayed intervention.

This case highlights several important points. Monoclonal immunotactoid glomerulopathy or glomerulonephritis with organized microtubular monoclonal immunoglobulin deposits should be considered even when initial serum and urine monoclonal studies are negative, because kidney biopsy may provide the first decisive evidence of a monoclonal process.^1^, ^2^, ^3^ In addition, even when the underlying B-cell clone does not initially meet conventional hematologic treatment criteria,^4^ progressive kidney injury may justify repeated reassessment and timely clone-directed therapy.^5^ Finally, effective clone-directed therapy may be accompanied by morphologic regression of the defining lesion, whereas delayed intervention may still permit irreversible chronic damage to accrue.

## Disclosure

MY and DK received research grant support from the Japan Society for the Promotion of Science (JSPS; KAKENHI). MM received honoraria from Chugai Pharmaceutical, Astellas, Kyowa Kirin, Mitsubishi Tanabe, Mochida Pharmaceutical, AstraZeneca, Bayer Yakuhin, Torii Pharmaceutical, Fuso Pharmaceutical Industries, Daiichi Sankyo Company, Teijin Pharma, Eli Lilly Japan, Nippon Boehringer Ingelheim, Kissei Pharmaceutical, and Kowa Company; research funding from Chugai Pharmaceutical and Kyowa Kirin; and scholarship donations from Kyowa Kirin, Torii Pharmaceutical, Fuso Pharmaceutical Industries, and Kissei Pharmaceutical. The other authors declared that they have no competing interests.

### Declaration of AI and AI-Assisted Technologies in the Writing process

During the preparation of this manuscript, the authors used ChatGPT (OpenAI) solely to assist with translation from Japanese into English and refinement of English phrasing. The authors independently reviewed and edited all output and take full responsibility for the final content of the manuscript.

## Supplementary Material

Supplementary file (PDF) **Supplementary Methods.** Kidney biopsy evaluation and laser microdissection–liquid chromatography–mass spectrometry. **Table S1.** Laboratory data over the clinical course. **Table S2.** Staining and antibody information. **Table S3.** Proteomic findings by laser microdissection–liquid chromatography–mass spectrometry.

## Footnotes

Supplementary file (PDF)
**Supplementary Methods.** Kidney biopsy evaluation and laser microdissection–liquid chromatography–mass spectrometry. **Table S1.** Laboratory data over the clinical course. **Table S2.** Staining and antibody information. **Table S3.** Proteomic findings by laser microdissection–liquid chromatography–mass spectrometry.
