# Supplementary material for: Disappearance of the Defining Organized Ultrastructural Lesion in Chronic Lymphocytic Leukemia-Associated Monoclonal Immunotactoid Glomerulopathy After Ibrutinib Therapy
Source: Kidney Int Rep. 2026 Jun 3;11(8):106643. doi: 10.1016/j.ekir.2026.106643 (PMC13333286; doi:10.1016/j.ekir.2026.106643)
Supplement: Supplementary file (PDF) — Supplementary Methods. Kidney biopsy evaluation and laser microdissection–liquid chromatography–mass spectrometry. Table S1. Laboratory data over the clinical course. Table S2. Staining and antibody information. Table S3. Proteomic findings by laser microdissection–liquid chromatography–mass spectrometry. [file mmc1.docx]

**Supplementary Methods**

**Kidney biopsy evaluation and laser microdissection–liquid chromatography–mass spectrometry**

**Kidney biopsy evaluation**

Kidney biopsy specimens were evaluated by light microscopy, immunofluorescence, immunohistochemistry, and electron microscopy using standard diagnostic renal pathology protocols.　For light microscopy, formalin-fixed paraffin-embedded kidney biopsy sections were stained with hematoxylin and eosin, periodic acid–Schiff, periodic acid–methenamine silver, Masson trichrome, and Congo red. Direct immunofluorescence was performed on frozen kidney biopsy sections using antibodies against IgG, IgA, IgM, C3, C1q, κ light chain, λ light chain, and IgG subclasses. Staining intensity was assessed semiquantitatively as part of routine diagnostic renal pathology evaluation. Available staining and antibody information is summarized in Supplementary Table S2.　DNAJB9 immunohistochemistry was performed on formalin-fixed paraffin-embedded kidney biopsy sections at an external institution using a validated diagnostic protocol to evaluate the possibility of fibrillary glomerulonephritis ^(S1,S2)^. Antibody information for DNAJB9 immunohistochemistry is provided in Supplementary Table S2. Electron microscopy was performed using kidney biopsy tissue processed according to standard diagnostic protocols.

**Laser microdissection and liquid chromatography–mass spectrometry**

Laser microdissection followed by liquid chromatography–mass spectrometry was performed on glomerular tissue obtained from the first kidney biopsy at an external institution. Mass spectrometry data were acquired using data-independent acquisition and analyzed using DIA-NN, an open-source software platform.

The analyzed glomerular area was limited to 240,000 μm², which was below the recommended area of 600,000 μm²; therefore, the proteomic findings were interpreted with caution.

The analysis detected more peptides derived from the variable region of the κ light chain than from λ-derived peptides, consistent with the κ-dominant pattern observed by immunofluorescence. Detailed proteomic findings are provided in Supplementary Table S3.

**Supplementary Table S1**

**Laboratory data over the clinical course**

**Supplementary Table S2**

**Staining and antibody information**

**Supplementary Table S3**

**Proteomic findings by laser microdissection–liquid chromatography–mass spectrometry**

**Supplementary References**

S1. Nasr SH, Vrana JA, Dasari S, et al. DNAJB9 is a specific immunohistochemical marker for fibrillary glomerulonephritis. *Kidney Int Rep*. 2018;3(1):56-64.

S2. Klomjit N, Alexander MP, Zand L. Fibrillary glomerulonephritis and DnaJ homolog subfamily B member 9 (DNAJB9). *Kidney360*. 2020;1(9):1002-1013.
